# Predictive factors for efficacy of oxaliplatin-based chemotherapy in advanced well-differentiated neuroendocrine tumors: an observational cohort study and meta-analysis

**DOI:** 10.3389/fendo.2025.1595151

**Published:** 2025-05-14

**Authors:** Jian Wang, Xiangling Wang, Yunxia Chu, Shuguang Li, Jing Hao

**Affiliations:** Department of Medical Oncology, Qilu Hospital of Shandong University, Jinan, China

**Keywords:** well-differentiated neuroendocrine tumors, oxaliplatin, pancreatic, Ki-67 index, MGMT, hepatic tumor burden

## Abstract

**Background:**

Oxaliplatin-based chemotherapy (OX-CT) has shown promising antitumor activity in advanced well-differentiated neuroendocrine tumors (WD-NETs). However, no meta-analysis has been conducted to explore the factors associated with ORR and PFS of OX-CT, and data are still limited in Chinese cohort.

**Methods:**

We performed a retrospective cohort study with advanced WD-NETs who received OX-CT. We also conducted a systematic review and performed a meta-analysis to explore factors associated with ORR and PFS.

**Results:**

A total of 27 patients were included, with 21 receiving OX-CT as first line. Furthermore, 18 were of pancreas origin, and the median Ki-67 was 30%. The ORR and DCR were 29.6% and 81.5%, respectively. The median PFS was 9.3 months (95%CI: 4.6–14.0), and OS was not reached. A Ki-67 value >10% predicted higher ORR (36.4% vs. 0.0%, *p* = 0.28) and better PFS (10.0 vs. 2.1 months, *p* = 0.06). Patients with hepatic tumor burden ≤25% had a similar ORR (33.3% vs. 22.2%, *p* = 0.68), but with a trend of longer PFS (10.2 vs. 4.7 months, *p* = 0.21) than those >25%. Both ORR and PFS were independent of MGMT status. A total of 962 patients were included in the systemic review. The pooled ORR (28.2%, *p* = 0.84) and DCR (82.9%, *p* = 0.85) were comparable with this cohort. No difference was observed between GEMOX and FOLFOX/CAPOX in both ORR (23.9% vs. 29.6%, *p* = 0.19) and PFS (10.5 vs. 11.8 months, *p* = 0.69). Enhanced ORR was seen in pNETs than epNETs (36.8% vs. 16.7%, *p* < 0.001) and also in G3 NETs than G1–2 NETs (45.5% vs. 24.7%, *p* < 0.001). The pooled median PFS and OS were 10.8 months (95%CI: 8.8–12.8) and 30.4 months (95%CI: 24.8–35.9).

**Conclusions:**

Oxaliplatin-based chemotherapy could be a good option for advanced WD-NETs with high Ki-67 index and pancreatic origin.

## Introduction

Neuroendocrine neoplasms (NENs) are a heterogeneous group of malignancies originating from the neuroendocrine cells of various organs. Most NENs are well-differentiated neuroendocrine tumors (WD-NETs), and more than 50% of NETs arise in the gastrointestinal tract and pancreas (1). For unresectable or metastatic NENs, systemic therapeutic options have been proven to delay disease progression and improve survival, which include somatostatin analogs (SSAs), inhibitors of the mammalian target of rapamycin, receptor tyrosine kinase inhibitors, peptide receptor radionuclide therapy (PRRT), liver targeted therapy, and cytotoxic agents (2). Belzutifan, a selective small-molecule inhibitor of hypoxia-inducible factor 2a, has demonstrated a high response rate in VHL-associated pancreatic neuroendocrine tumors and also recently approved by the US Food and Drug Administration (3). Given the high heterogeneity of WD-NETs and the lack of robust predictive biomarkers to guide treatment selection, no clear recommendations exist regarding therapeutic sequences or in combination.

Currently, the role of cytotoxic chemotherapy in advanced WD-NETs remains debated and predominantly considered in advanced NETs with high Ki-67 proliferation index and tumor burden, in a rapidly progressive disease, after failure of the other therapies, and/or when tumor size reduction by cytotoxic intervention is a therapeutic goal (4–6). So far, alkylating agents alone, such as streptozotocin, temozolomide, dacarbazine, or in combination with 5-fluorouracil or capecitabine, are widely used (7). However, alkylating agent-induced DNA damage can be repaired by O^6^-methylguanine-DNA methyltransferase (MGMT), and MGMT positivity was shown to be a relevant biomarker for poor efficacy (8, 9). The wider molecular analysis revealed that no particular mutational or transcriptional profile was associated with TMZ response in contrast to MGMT promoter methylation (10).

Oxaliplatin-based chemotherapy (OX-CT) has also shown promising antitumor activity across multiple treatment lines, with response rates ranging from 17% to 30% and progression-free survival ranging between 7 and 14 months (4–6), which was not influenced by the MGMT status (11–13). Moreover, the addition of bevacizumab to FOLFOX yielded a higher ORR of 52.6% in pancreatic NETs and 56.5% in grade 3 NETs (14). These evidence-based findings have been incorporated into the updated ENETS guidance papers in digestive neuroendocrine tumors, especially in high Ki-67 index or grade 3 differentiation (15–17). Despite the fact that more and more prospective and retrospective evidence have emerged, no meta-analysis has been done to address oxaliplatin-based chemotherapy in advanced WD-NETs and potential factors to predict response. Moreover, clinical data in Chinese cohort is still lacking.

## Methods

### Cohort study design and patients

This retrospective observational cohort study enrolled patients (pts) with locally advanced or metastatic WD-NETs who were consecutively treated with oxaliplatin-based chemotherapy from June 2020 to July 2024 in Qilu Hospital cancer center of Shandong University.

Patients who were eligible were required to meet the following criteria: (1) age ≥18, (2) ECOG performance status ≤2, (3) histologically confirmed NETs, locally advanced or metastatic, well-differentiated, and grade 2 or 3 according to the World Health Organization 2019 classification, (4) have received oxaliplatin-based regimens at any line, FOLFOX/CAPOX/SOX with or without bevacizumab, (5) known MGMT status, and (6) at least one measurable target lesion. The key exclusion criteria were poorly differentiated neuroendocrine carcinomas (NEC).

The study was conducted in accordance with the Declaration of Helsinki (as revised in 2013) and was approved by the Ethics Committee of Qilu Hospital of Shandong University (2015078).

### Treatment

CAPOX consisted of 130 mg/m^2^ oxaliplatin on day 1 and capecitabine at a dose of 750–1,000 mg/m^2^ twice daily for 2 weeks. SOX consisted of 130 mg/m^2^ oxaliplatin on day 1 and S-1 (tegafur, gimeracil, and oteracil potassium capsule) at a dose of 40–60 mg twice daily for 2 weeks. The treatment course was repeated every 3 weeks.

The mFOLFOX6 regimen was administered as 85 mg/m^2^ of oxaliplatin and 200 mg/m^2^ of levo-leucovorin over 2 h, followed by a 400 mg/m^2^ bolus of fluorouracil and then a 2,400 mg/m^2^ bolus of fluorouracil by a 46 h infusion repeated every 2 weeks.

Bevacizumab was administered at 5 mg/kg every 14 days or 7.5 mg/kg every 21 days.

Fluorouracil or capecitabine/S-1 **±** bevacizumab maintenance therapy was allowed for patients who achieved a stable disease or response. At first progression, re-introduction with all of the drugs was recommended.

The patients received routine supportive care at the discretion of the treating physician. Standard dose adjustment criteria were applied to both CAPOX and mFOLFOX6.

### Immunochemistry of MGMT

The nuclear expression of the MGMT protein was assessed by immunohistochemistry (18) using paraffin-embedded sections with a mouse monoclonal MGMT antibody (ZM-0461, ZSGB-BIO). MGMT expression was assessed on a whole slide using a score based on nuclear staining intensity (0–3) multiplied by the proportion of stained cells (0%–100%). The score ranges from 0 to 300. Deficient MGMT (dMGMT) was defined if the score ≤50, while proficient MGMT (pMGMT) was those with score >50.

### Hepatic tumor burden evaluation

The quantified hepatic tumor load came from six slices of a CT/MRI scan with the most amount of disease by a semi-quantitative three-dimensional approach (19). Hepatic tumor burden (HTB) was categorized as 0%, more than 0% but 10%, more than 10% but 25%, more than 25% but 50%, or more than 50%.

### Cohort study endpoints and statistical analysis

The primary endpoint was the objective response rate (ORR). The secondary endpoints were progression-free survival (PFS), overall survival (OS), disease control rate (DCR), and safety. Tumor response was evaluated by imaging studies (computed tomography or magnetic resonance imaging) in accordance with the Response Evaluation Criteria in Solid Tumors (RECIST 1.1). Imaging studies were performed at baseline and were repeated every 8–12 weeks (the exact interval was at the physician’s or the patient’s discretion). PFS was calculated from the first day of the first cycle of chemotherapy cycle until clinical and/or radiological progression. All of the patients were followed up for survival. OS was defined as the time interval from the date of the first cycle of chemotherapy until death from any cause. Adverse events were recorded according to Common Terminology Criteria for Adverse Events (version 5.0) of the National Cancer Institute.

Baseline characteristics and toxicities were assessed using a descriptive method. Efficacy analyses were restricted in patients who had completed at least two treatment cycles and one response evaluation. The Clopper–Pearson method was used to calculate the 95% confidence intervals for ORR and DCR. Fisher exact test was used to calculate the *p*-values of ORR difference between groups. PFS and OS curves were obtained by the Kaplan–Meier method, and data were reported with two-sided 95% confidence intervals (CI). Statistical significance was set at *p <*0.05. Exploratory *post-hoc* subgroup analyses of PFS were performed using log rank method. All analyses were performed using SPSS software (version 26.0) and R version 4.3.2.

### Systemic review and meta-analysis: Search strategy, eligibility criteria, data extraction, and statistical analysis

The systematic review and meta-analysis were performed according to the PRISMA (Preferred Reporting Items for Systematic Reviews and Meta-analysis) guidelines (20). The literature search in PubMed, Embase, and Cochrane Central Register of Controlled Trials was conducted from January 2000 to December 2024 to identify available studies, both published and in abstract form, that evaluated the efficacy of oxaliplatin-based chemotherapy for the treatment of advanced well-differentiated neuroendocrine tumors (NETs). The search strategy was as follows: (“oxaliplatin” or “L-OHP” or “OXA”) and (“neuroendocrine tumors” or “NETs” or “neuroendocrine neoplasm” or “NEN” or “carcinoid tumor”). Abstracts on NENs from several important international meetings including American Society of Clinical Oncology (ASCO), European Society for Medical Oncology (ESMO), European Neuroendocrine Tumor Society (ENETS), and North American Neuroendocrine Tumor Society (NANETS) from January 2018 to December 2024 were checked to identify potentially relevant studies.

The following criteria were used to identify eligible data, studies describing oxaliplatin-based chemotherapy in advanced WD-NETs and reporting tumor efficacy outcomes, including ORR, PFS, OS, and safety. In the screened studies, if well-differentiated neuroendocrine tumors (WD-NETs) and poorly differentiated neuroendocrine carcinomas (NEC) were mixed together, only the data of WD-NETs was extracted. If it was impossible to distinguish between WD-NETs and NEC, the literature would be excluded. The search was limited to phase I–III prospective clinical trials and retrospective studies including more than 10 patients. Meta-analyses, editorials, commentaries, case reports and case series, and review articles were excluded. The selected studies and abstracts were independently evaluated by two authors (JW and XW); any disagreement was resolved through discussion and negotiation.

The following data were extracted and entered into a standardized, predesigned Excel form: (1) study ID, author, publication year, and study design; (2) site of primary tumor and histological grading; (3) total number of patients; (4) oxaliplatin-based regimens; (5) lines of treatment; (6) ORR, DCR, median PFS, and OS; (7) duration of treatment; and (8) adverse effects.

The pooled ORR, DCR, median PFS, and median OS were calculated and weighted using generic inverse variance in a random-effects model by STATA 17.0 as well as subgroup analysis for patients with pancreatic NETs (pNETs) and extra-pancreatic NETs (epNETs), G1–2 and G3 NETs, and chemotherapy regimens (GEMOX vs. fluoropyrimidine-based chemotherapy). *I* ^2^ was used as the indicator of heterogeneity. *I* ^2^ <25%, 25% ≤*I* ^2^ <50%, and *I* ^2^ ≥50% indicated low, moderate, and high heterogeneity, respectively. Chi-square test was used to calculate the p-values of ORR and DCR and log rank test for PFS and OS. *p <*0.05 was considered to be statistically significant.

In order to assess the impact of studies with low quality, a sensitivity analysis was performed by repeating the meta-analysis, excluding those studies with a score less than 7 using JBI PACES.

## Results

### Patients’ characteristics of cohort study and meta-analysis

A total of 27 patients were enrolled in this cohort study between June 2020 and July 2024, and the median follow-up was 20.7 months. The clinico-pathological characteristics are summarized in **Table 1**. The median age was 56 (25–70) years. Furthermore, 18 patients were male. All patients were non-functional. The primary tumor was resected in 12 patients, and 18 patients were of pancreatic origin. The median Ki-67 index was 30% (range 3%–60%), with nine and 18 classified as G2 and G3. MGMT was positive in 18 (66.7%) patients. Liver metastases were diagnosed in 22 patients (81.5%), with hepatic tumor burden ≤10%,10%–25%, 25%–50%, and >50% being 48.1%, 18.5%, 7.4%, and 25.9%, respectively. Oxaliplatin-based chemotherapy was administrated as first-line treatment in 19 (70.4%) cases. Five patients received concurrent bevacizumab or other anti-angiogenic drugs. The median number of treatment cycles was six, ranging from one to 12. Two patients had reintroduction of OX-CT after a treatment-free interval of 9.3 and 19.7 m, respectively.

**Table 1:** Patients’ features at baseline according to primary location.

Characteristics	Total (*n* = 27)	pNET (*n* = 18)	epNET (*n* = 9)	*p*
Age				
Median age, (range)	56 (25–70)	53.5 (24–67)	65 (39–70)	0.042
Sex no. (%)				
Male	18 (66.7)	12 (66.7)	6 (66.7)	1
Female	9 (33.3)	6 (33.3)	3 (33.3)	
Performance status no. (%)				
0	10 (37.0)	7 (38.9)	3 (33.3)	0.778
1	17 (63.0)	11 (61.1)	6 (66.7)	
Primary tumor location, no. (%)				
Pancreas	18 (66.7)	18 (100.0)		NA
Rectum	4 (14.8)		4 (44.4)	
Stomach	2 (7.4)		2 (22.2)	
Duodenum	1 (3.7)		1 (11.1)	
Liver	1 (3.7)		1 (11.1)	
Unknown	1 (3.7)		1 (11.1)	
WHO grade, no. (%)				
NET/atypical grade 2	9 (33.3)	5 (27.8)	4 (44.4)	0.423
NET G3	18 (66.7)	13 (72.2)	5 (55.6)	
Ki-67, %, median (range)	30 (3–60)	40 (6–80)	25 (3–50)	0.101
Previous lines of systemic treatment, no. (%)				
0	20 (74.1)	12 (66.7)	8 (88.9)	0.438
1	6 (22.2)	5 (27.8)	1 (11.1)	
2	1 (3.7)	1 (5.6)		
Metastatic pattern, no. (%)				
Locally advanced disease	4 (14.8)	4 (22.2)		0.179
Only liver metastases	11 (40.7)	8 (44.4)	3 (33.3)	
Liver + extrahepatic metastases	11 (40.7)	6 (33.3)	5 (55.6)	
Extrahepatic metastases	1 (3.7)		1 (11.1)	
Location of metastasis, no. (%)				
Liver	22 (81.5)	14 (77.8)	8 (88.9)	0.333
Lung	2 (7.4)	1 (5.6)	1 (11.1)	
Lymph node	10 (37.0)	6 (33.3)	4 (44.4)	
Bone	3 (11.1)	1 (5.6)	2 (22.2)	
Others	3 (11.1)	2 (11.1)	1 (11.1)	
Somatostatin receptors expression, no. (%)				
Positive	20 (74.1)	13 (72.2)	7 (77.8)	0.038
Negative	5 (18.5)	5 (27.8)		
Unknown	2 (7.4)		2 (22.2)	
MGMT protein expression by IHC, no. (%)				
Positive	18 (66.7)	12 (66.7)	6 (66.7)	1
Negative	9 (33.3)	6 (33.3)	3 (33.3)	
Hepatic tumor burden, no. (%)				
≤10%	13 (48.1)	8 (44.4)	5 (55.6)	0.831
10%–25%	5 (18.5)	4 (22.2)	1 (11.1)	
25-%–50%	2 (7.4)	1 (5.6)	1 (11.1)	
>50%	7 (25.9)	5 (27.8)	2 (22.2)	
Type of chemotherapy, no. (%)				
CAPOX	20 (74.1)	13 (72.2)	7 (77.8)	0.038
FOLFOX	5 (18.5)	5 (27.8)		
SOX	2 (7.4)		2 (22.2)	

MGMT, O^6^-methylguanine-DNA methyltransferase; CAPOX, capecitabine and oxaliplatin; FOLFOX, oxaliplatin plus 5-fluorouracil; SOX, S-1 and oxaliplatin.

As shown in **Supplementary Figure S1**; **Supplementary Table S1**, 17 selected articles and a total of 962 patients were included in the systemic review, which comprised 14 retrospective studies (11, 14, 21–32) and three phase II studies (13, 33, 34). Moreover, 434 pts received FOLFOX (oxaliplatin plus 5-Fluorouracil), 117 pts CAPOX (oxaliplatin plus capecitabine), 168 pts GEMOX (gemcitabine plus oxaliplatin), and 138 pts FOLFOX/CAPOX plus bevacizumab. Moreover, 48.2% (441/962) patients were pNETs, and G3 NETs accounted for 25.6% (246/962).

### Comparative response evaluation of this cohort and meta-analysis

**Figure 1:**
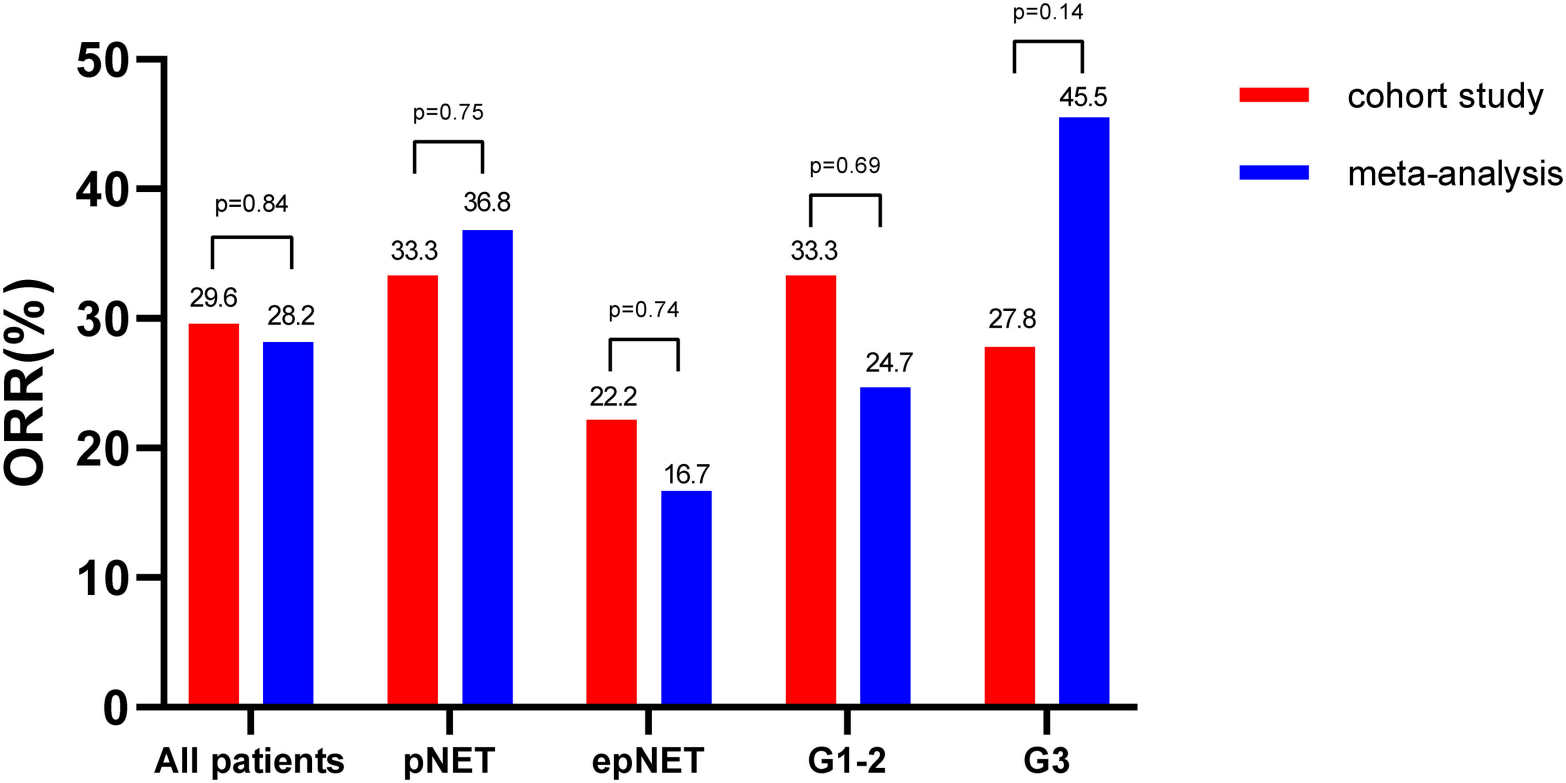
Comparative response analysis of this cohort and meta-study.

**Figure 2:**
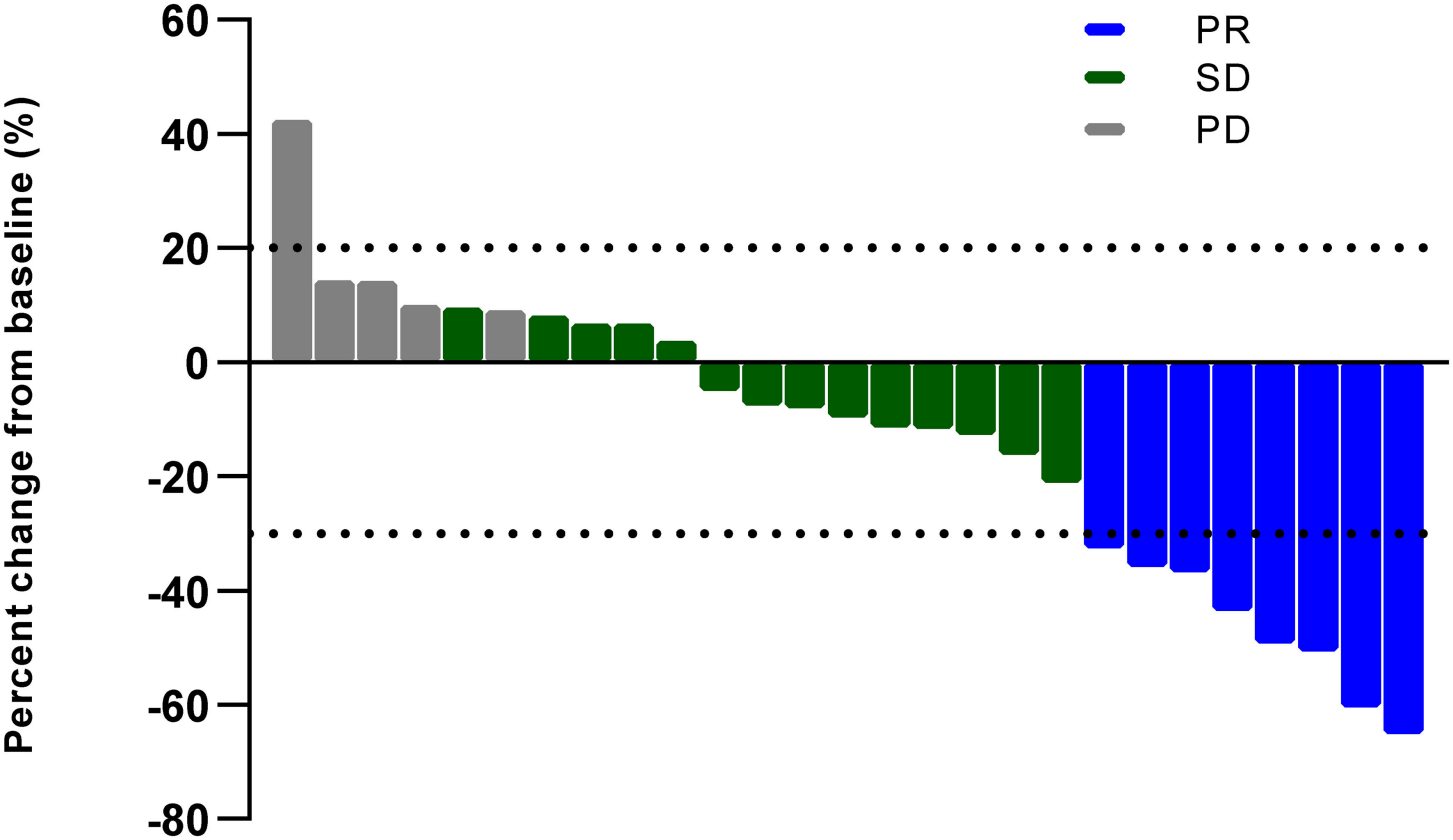
Best percent change in size of target lesions (PR, partial response; SD, stable disease; PD, progressive disease).

As shown in **Figures 1**, **2**, all of the patients in this cohort had at least one response evaluation, eight (29.6%, 95%CI: 13.8%–50.2%) pts achieved partial response, 14 (51.9%) had stable disease, and five (18.5%) had progression. The DCR was 81.5% (95%CI: 61.9%–93.7%). Both the ORR and DCR in the cohort were comparable to the pooled efficacy in meta-analysis (**Figure 1**; **Supplementary Figure S2**; **Supplementary Table S2**). The pooled ORR and DCR were 28.2% (95%CI: 24.0–32.4, *p* = 0.84) and 82.9% (95%CI: 78.9–86.8, *p* = 0.85).

In terms of impact of location on efficacy, pancreatic NETs showed a trend of higher ORR (33.3% vs. 22.2%, *p* = 0.45) than extra-pancreatic NETs in this cohort. In the meta-analysis, pNETs showed significantly higher ORRs (36.8%,129/348, 95%CI: 31.3–42.2) than epNETs (ORR: 16.7%, 73/407, 95%CI: 13.1–20.3, *χ* ^2^ = 35.0, *p <*0.01). Of note, the ORRs in pNETs (33.3% vs. 36.8%, *p* = 0.75) and epNETs (22.2% vs. 16.0%, *p* = 0.72) from this cohort and meta-analysis were consistent.

With regard to the impact of grade, G2 NETs in this cohort had similar ORR (33.3% vs. 27.8%, *p* = 0.55) with G3 NETs. By contrast, in this meta-analysis, G3 NETs was more sensitive to oxaliplatin-based chemotherapy with ORRs at 45.5% (61/132, 95%CI: 33.6–57.5), while G1–2 NETs showed a modest response rate (24.7%, 33/133, 95%CI: 13.3–36.0, *χ* ^2^ = 13.3, *p* < 0.01).

Sensitivity analysis was performed excluding those studies with low-quality (six studies with score less than 7 using JBI PACES and studies which did not discriminate pNETs from epNETs or G3 from G1–2 were excluded). The ORR difference between pNETs and epNETs (36.6% vs. 17.3%, *p* < 0.01) and G3 NETs and G1–2 NETs (43.2% vs. 24.7%, *p* < 0.01) remained statistically significant (**Supplementary Figure S3**).

### Survival outcome of this cohort and meta-analysis

**Figure 3:**
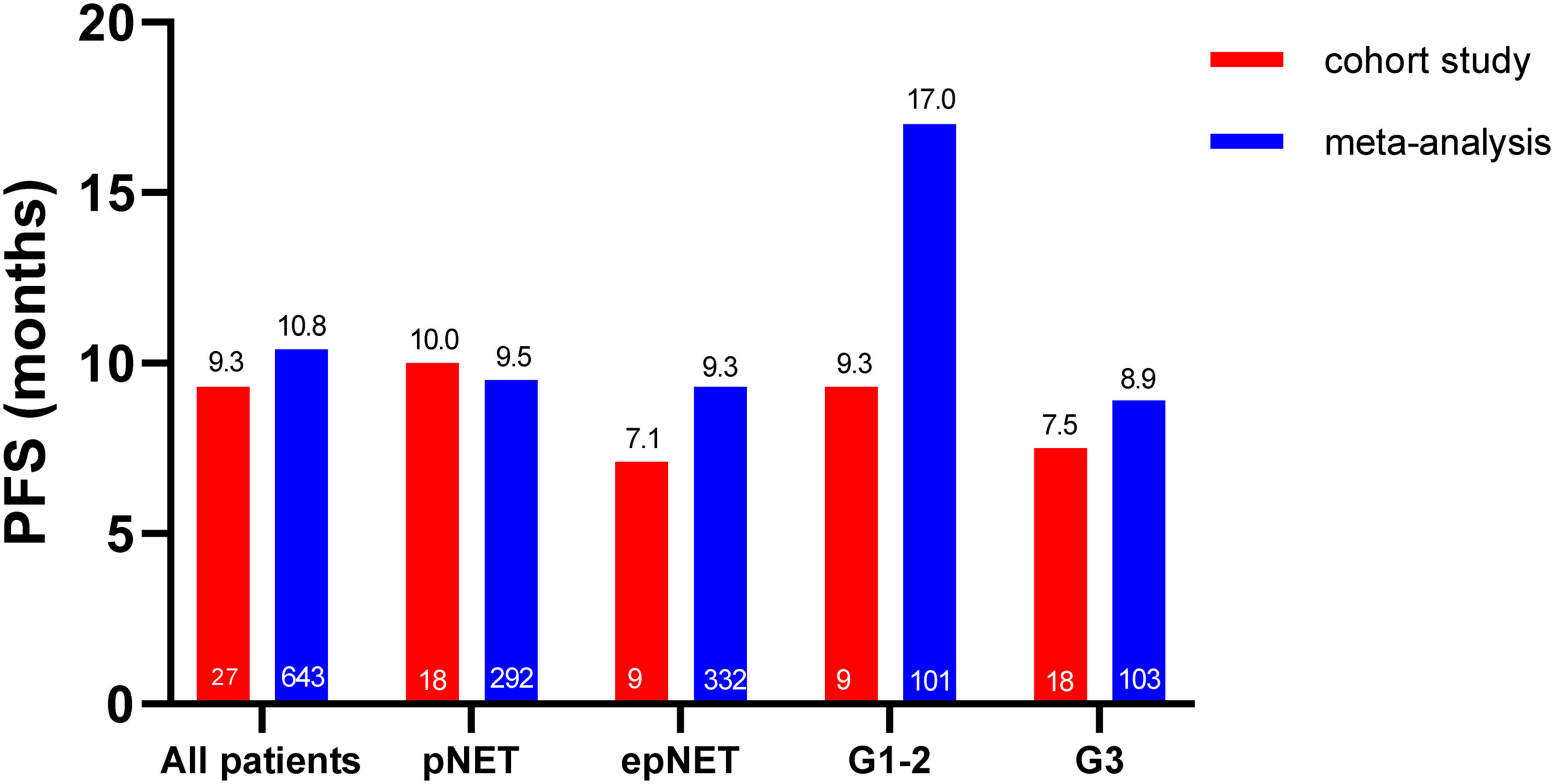
Comparative PFS analysis of this cohort and meta-analysis.

**Figure 4:**
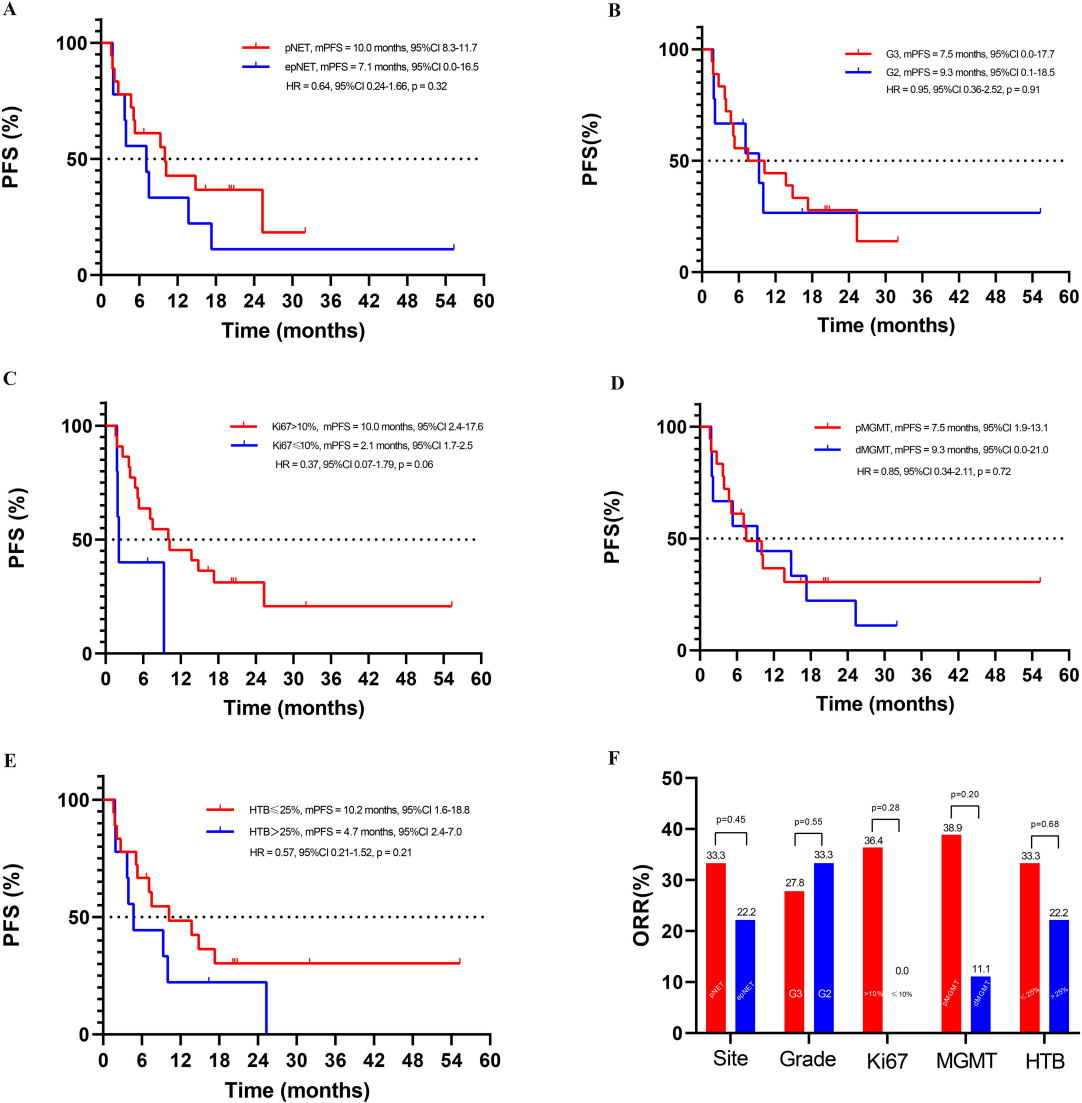
Exploratory subgroup analyses in this cohort study: **(A)** PFS of pNET and epNET, **(B)** PFS of G3 and G2 NET, **(C)** PFS based on Ki-67 index, **(D)** PFS based on MGMT status, **(E)** PFS based on HTB (hepatic tumor burden), **(F)** ORR of different subgroups.

As shown in **Figures 3**, **4**, the median PFS in this cohort was 9.3 months (95%CI: 4.6–14.0). No difference was observed between pNETs and epNETs (10.0 months, 95%CI: 8.3–11.7 vs. 7.1 months, 95%CI: 0.0–16.5, *p* = 0.32). G2 NETs had similar PFS (9.3 months, 95%CI: 0.1–18.5 vs. 7.5 months, 95%CI: 0.0–17.7, *p* = 0.91) with G3 NETs (7.5 months, 95%CI: 0.0–17.7, *p* = 0.91). The median OS was not reached.

In this meta-analysis, the pooled median PFS and OS were 10.8 months (95%CI: 8.8–12.8) and 30.4 months (95%CI: 24.8–35.9), respectively. The median PFS in pNETs was 9.5 months (*n* = 292, 95%CI: 7.1-11.9), which was similar to 9.3 months in epNETs (*n* = 332, 95%CI: 6.6–12.1, *p* = 0.59), while the G1-G2 NET (*n* = 101, 17.0 months, 95%CI: 12.5–21.4) had a much longer median PFS than G3 NET (*n* = 103, 8.9 months, 95%CI: 5.8–11.9, *p* = 0.04) (**Supplementary Figure S4**). Due to the lack of individual data in the meta-analysis, both the PFS and OS were not compared with this cohort.

Of five patients who received concurrent anti-angiogenic drugs, all had stable diseases and one case had radical operation after conversion chemotherapy. The PFS were 4.7, 7.1, 14.8, 20.4+, and 20.8+ months, respectively.

### Exploratory subgroup analyses in this cohort study: Ki-67 index, hepatic tumor burden, and MGMT status

As depicted in **Figure 4**, when 10% was used as the cutoff of Ki-67, higher ORR (36.4% vs. 0.0%, *p* = 0.28) and better PFS (10.0 months, 95%CI: 2.4–17.6 vs. 2.1 months, 95%CI: 1.7–2.5, *p* = 0.06) were seen in patients with Ki-67 >10% than those ≤10%.

Patients with hepatic tumor burden ≤25% showed a trend of longer PFS (10.2 months, 95%CI: 1.6–18.8 vs. 4.7 months, 95%CI: 2.4–7.0, *p* = 0.21) and similar ORR (33.3% vs. 22.2%, *p* = 0.68) when compared with liver tumor burden >25%.

The ORR in patients with positive MGMT expression was 38.9%, while it was 11.1% (*p* = 0.20) in the negative counterpart. Moreover, no impact of MGMT status was seen on PFS, with 7.5 months (95%CI: 1.9–13.1) and 9.3 months (95%CI: 0.0–21.0, *p* = 0.72) in pMGMT and dMGMT patients.

### Exploratory analyses of difference between chemotherapy regimens (GEMOX vs. FOLFOX/CAPOX)

**Figure 5:**
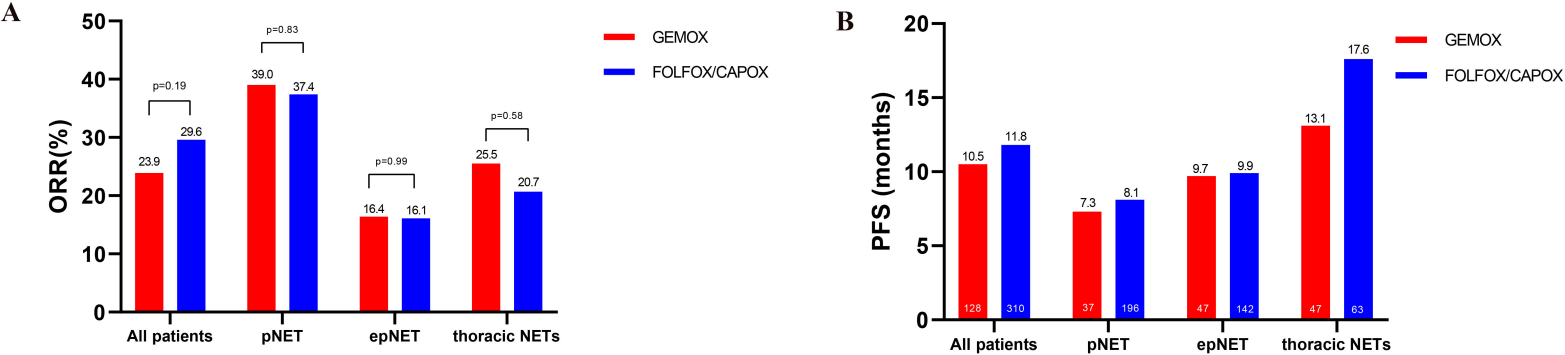
Comparative efficacy analysis of GEMOX and FOLFOX/CAPOX chemotherapy in the meta-analysis. **(A)** ORR, **(B)** PFS.

A total of 168 patients (17.5%) received GEMOX, and 133 (13.8%) patients could be used for efficacy analysis, while all the others were combined as fluoropyrimidine-based chemotherapy (FOLFOX/CAPOX). As shown in **Figure 5**, **Supplementary Figure S5**, the pooled ORR of patients treated with the GEMOX was 23.9% (32/133, 95%CI: 16.7–31.1) in meta-analysis compared to 29.6% (208/701, 95%CI: 24.4–34.8, *χ* ^2^ = 1.7, *p* = 0.19) in patients who received FOLFOX/CAPOX, indicating no statistical difference between the two regimens. Similarly, the median PFS was comparable between the two groups, with 10.5 months (95%CI: 2.9–18.1) in the GEMOX group versus 11.8 months (95%CI: 8.8–14.8, *p* = 0.69) for FOLFOX/CAPOX.

In terms of impact of both location and regimens on efficacy, as shown in **Figure 5**, both the ORR and PFS were comparable in the two groups (GEMOX vs. FOLFOX/CAPOX), either in pancreatic NETs and extra-pancreatic NETs.

Furthermore, 25.0% (61/244) and 36.1% (48/133) patients were of thoracic origin, respectively, in FOLFOX/CAPOX and GEMOX groups. In thoracic NETs, the ORR to GEMOX was 25.5% (95%CI: 13.1–38.0), while it was 20.7% to FOLFOX/XELOX (95%CI: 2.7–38.6, *p* = 0.58). The PFS was comparable between the two groups in thoracic NETs, with 13.1 months (95%CI: 6.7–19.5) in the GEMOX group versus 17.6 months (95%CI: 10.8–24.4, *p* = 0.21) for FOLFOX/CAPOX (**Supplementary Figure S6**).

### Adverse effects

Grade 3 or 4 treatment-related adverse events occurred in 18.5% patients, which was similar to the pooled side effects in this meta-analysis (24.2%, *p* = 0.32) summarized in **Supplementary Table S3**. The most common side effects of any grade in this cohort were peripheral sensory neuropathy (59.3%), nausea (37.0%), asthenia (33.3%), hand-foot syndrome (29.6%), and leukopenia (29.6%).

## Discussion

To our knowledge, this meta-analysis was the first comprehensive overview of the available data for oxaliplatin-based chemotherapy in well-differentiated advanced NETs. Albeit with a small size and different baseline clinicopathological characteristics from systemic review, our single-center cohort study in Chinese WD-NETs aligned with results of the meta-analysis and confirmed that oxaliplatin-based chemotherapy was an effective and safe therapeutic option. There were three major findings from both the meta-analysis and our study.

Firstly, oxaliplatin-based chemotherapy showed promising activity in WD-NETs. Notably, this therapeutic activity appears independent of both MGMT status and chemotherapy regimens (GEMOX or FOLFOX/CAPOX). With the exception of MGMT-NET trial, there is a paucity of direct comparative studies evaluating oxaliplatin-based regimens against the others, such as CAPTEM- and STZ-based chemotherapy. There was ongoing controversy regarding the optimal choice of treatment regimens.

The MGMT-NET study was the first randomized trial comparing alkylating agents and oxaliplatin-based chemotherapy in WD-NETs (13). In the total population with both dMGMT and pMGMT, oxaliplatin-based chemotherapy had a comparable activity to alkylating agents, with the best ORR of 30.2% vs. 35.5%, PFS of 12.6 vs. 12.1 m, and OS of 48.8 vs. 50.2 m. However, in proficient MGMT NETs, the best ORR was higher in oxaliplatin-based chemotherapy than alkylating agents (38.9% vs. 11.5%), albeit the PFS was not significantly different between chemotherapy arms. Of note, CAPTEM was the most commonly used alkylating regimen (50%) in the MGMT-NET trial, while STZ-fluorouracil accounted for only 4.8%. As representative of alkylating agents, most studies on STZ-based regimens focus on well-differentiated G1-G2 pancreatic NETs, with a median ORR of 33% and PFS of 14.5 m. However, data on G3 NETs and ep-NETs were inconclusive (35, 36). By far, streptozotocin was still unavailable in China.

In contrast with alkylating agents, the efficacy of oxaliplatin-based chemotherapy was independent of MGMT status (13). In the oxaliplatin arm of the MGMT-NET trial with MGMT status assayed by IHC (*n* = 31, 20 dMGMT and 11 pMGMT) (13), the 3-month ORR (35.0% vs. 27.3%) and PFS (12.2 and 12.4 months in dMGMT and pMGMT) were quite similar. Although there were more G3 (66.7%) and pMGMT (66.7%) NETs in our cohort, ORR of 29.6% was comparable to that of the oxaliplatin group in MGMT-NET.

Of note, the technique to determine the MGMT status remains an issue in clinical practice. In the two prospectively randomized trials (MGMT-NET and E2211), the concordance between methylation test (using pyrosequencing) and IHC was only 58.3% and 36%, respectively. However, both methodologies showed comparable predictive value for response to temozolomide. In addition, a low MGMT score (≤50) was found as a good cutoff to predict tumor response (18). Besides that, IHC was very convenient and cost-effective. Thus, in our study, the MGMT status was assessed using IHC, which aligns with the methodology described by Cros et al. (18) and in the MGMT-NET trial (13).

With regard to the impact of different oxaliplatin-based regimens, no significant difference in ORR and PFS, respectively, was found in patients who received GEMOX or fluoropyrimidine-based chemotherapy (FOLFOX/CAPOX) in this meta-analysis. Although there was a much higher percentage of patients receiving GEMOX in the oxaliplatin group of the MGMT-NET study than this meta-analysis (81.4% vs. 17.5%), the ORR was comparable (27.9% vs. 23.9%). This consistency suggested that oxaliplatin-based chemotherapy had a similar activity whether combined with gemcitabine or fluoropyrimidine. GEMOX was often used in thoracic NETs. Of note, 35.7% (15/42) in the MGMT-NET study and 36.1% (48/133) in the GEMOX group of this meta-analysis were of thoracic origin. Even when examining the subgroup of thoracic NETs, GEMOX maintained comparable efficacy to fluoropyrimidine-based chemotherapy in terms of both ORR and PFS.

Besides that, the growing use of the FOLFIRINOX (leucovorin, 5-FU, and oxaliplatin, irinotecan) schedule was recommended in NCCN guidelines (37) in NET G3 with unfavorable biology. Two cases of G3 NETs were included in the study by Borghesani et al. (38), with the promising ORR of 50% (1/2) to mFOLFIRINOX and PFS of 15.4 m, which appeared to confer a PFS advantage over our cohort and this meta-analysis. Future prospective trials are warranted to validate the potential superiority of mFOLFIRINOX in G3 NETs and to determine the optimal Ki-67 index cutoff for predicting treatment response.

Secondly, chemotherapy appears to elicit a greater antitumor activity in patients with pNETs in both our cohort and meta-analysis. The high sensitivity to chemotherapy for pNET was not only specific to oxaliplatin-based chemotherapy but also the same to alkylating agents (6, 11, 39–41). Although the significance of higher chemotherapy response in pNET was lost when low-quality studies were excluded in a sensitivity analysis, the effect was still suggestive (OR = 0.45, 95%CI: 0.19–1.07) and approaching the threshold for marginal significance (*p* = 0.07) (6). Of note, there were no thoracic NETs in non-pNETs in this systemic review, and all patients received non-oxaliplatin-based chemotherapy. Besides that, only 125 non-pNETs and 111 pNETs were included in the review (6). By contrast, a larger population (521 non-pNETs and 441 pNETs) was included in this meta-analysis. A significant lower ORR in the non-pNET patients when compared to pNETs was shown, which was independent of chemotherapy regimens (GEMOX vs. FOLFOX/CAPOX). The significance also remained after a sensitivity analysis excluding those studies with low quality.

By far, the reasons why well-differentiated pNETs (mainly G1-G2) were more sensitive to cytotoxic agents than epNETs were still unclear. MGMT methylation was ever reported to be significantly higher in pancreatic NET (50%) than extra-pancreatic NET (0%–15%) (18, 42–44), which may partly explain the high efficacy of CAPTEM. However, it is hard to be correlated with response to oxaliplatin. Actually, no difference was seen in MGMT expression between pNETs (66.7%) than epNETs (66.7%) in our study. Although pNETs have more frequent mutations in the MEN1 and DAXX/ATRX genes which were up to 44% and 40%, respectively (45, 46), these molecular alterations failed to predictive of chemotherapy response (10, 47). Considering the very heterogeneous epNETs, there should be a certain proportion of epNET patients for whom chemotherapy may be very active. Thus, specific patient selection criteria (yet to be identified) other than the primary location of the NET may be more important in therapeutic decision-making.

Thirdly, tumor grade and Ki-67 had an important impact on the response rate to oxaliplatin-based chemotherapy. In the meta-analysis, the ORR of OX-CT in G3 NET was very promising and significantly higher than that in G1–2 NET (45.5% vs. 24.7%). However, PFS was worse than G1–2 NET (8.9 vs. 17 m), reflecting a generally better prognosis in low proliferative tumors. By far, the prognosis of NET G3 patients is poor, and management is still challenging due to the lack of prospective data. Chemotherapy is still most commonly used in clinical practice. Among the different chemotherapy regimens, platinum-etoposide (PE) chemotherapy, which is the standard treatment in advanced neuroendocrine carcinoma, has shown to be less active in G3 NETs (24 cases) with ORR of 24% and PFS of only 5 months (48). In one retrospective analysis with NET G3 from three German cancer centers (27), the median PFS for PE chemotherapy (6.9 months) was much lower than non-PE (9.0 m). As for the optimal non-PE regimens, both alkylating agents and oxaliplatin-based chemotherapy were effective options. When used as first line, FOLFOX was shown to have a higher response rate than CAPTEM (56.4% vs. 27.3%), which might result from a higher Ki-67 index in the FOLFOX arm. However, when looking only at G3 NETs with pancreatic origin, the PFS for FOLFOX and CAPTEM were 8.5 and15.2 m, respectively. For extra-pancreatic G3 NETs, the PFS for CAPTEM and FOLFOX were 1.6 and 6.9 (27). All of the retrospective results indicated that both primary location and grade should be taken into consideration when making decision on chemotherapy regimens.

Apart from grade, our study also analyzed the specific Ki-67 range to predict response to oxaliplatin-based chemotherapy. We found that a Ki-67 cutoff of 10% can discriminate outcomes in G2-G3 NET. No objective response has been observed with FOLFOX/CAPOX in tumors with Ki-67 less than 10%, while patients with Ki-67 more than 10% had a response as high as 36.4%. Of note, in the 10 pancreatic NETs with Ki-67 ≥40%, the ORR was 40% (data not shown). By contrast, in the study by Lamberti, none of the 34 patients with Ki-67 ≥40% who received FOLFOX/CAPOX (34 cases) had a response (49). Such a discrepancy on Ki-67 as a predictive marker was also seen in CAPTEM. One retrospective multicenter study by Wang et al. indicated that patients with a Ki-67 range of 10%–40% were the most responsive to the CAPTEM regimen (50). In one study by Jeong et al. for patients with Ki-67 >20% and <30% versus ≥30% and <55% versus ≥55%, the ORR values of CAPTEM were 18.2% versus 50.0% versus 0% (*p* = 0.079), respectively (51). MGMT was reported to be less frequently inactivated in NET G3 than G1/G2 (13, 52, 53), which might underlie the different Ki-67 range to predict response to alkylating agents. In our study, more deficient MGMT pts were in G2 NETs (43.4%) than G3 (27.8%), and only one patient (1/9) with Ki-67 more than 40% was MGMT-negative. Thus, for the heterogenous G3 NETs, distinct subgroups might benefit from alkylating agents and oxaliplatin-based chemotherapy, respectively.

In our cohort study, hepatic tumor burden was also predictive to the efficacy of oxaliplatin-based chemotherapy. The median PFS in patients with hepatic tumor burden ≤25% (10.2 months) was longer than those with >25% (4.7 m). The response rate in patients with low HTB also had a trend of higher ORR than those with high HTB. Hepatic tumor burden was known as a prognostic factor in neuroendocrine neoplasms. Low HTB was associated with prolonged survival and favorable treatment response (54–56).

Several limitations of our study deserve comments. Firstly, most studies in systemic review were of retrospective design, small sample size and mixed population, different treatment cycles, and both progressive or stable disease at enrollment. Hence, selection bias was inevitable, and statistical power for subgroup analyses was limited, so did our study in a Chinese cohort. Future prospective studies are warranted to extend these findings. Secondly, the relevance of ORR has been challenged in the field of NET for a long time. The response rate often rose as Ki-67 grade increased, while overall survival decreased. Similarly, the better ORR in G3 than G1–G2 to oxaliplatin-based regimens in this review did not transform significantly to the advantages of disease control, PFS, and OS. Increasingly, many trials involving NETs have evolved from the overall response rate to use PFS (ECOG-ACRIN E2211, NCT04919226, NCT03351296, and NCT02595424) or OS (NCT04365023) as the primary endpoint. Thirdly, the included studies were published between 2007 and 2024. The WHO classification of NETs was updated during this period, leaving the analysis of G3 NET inaccurate in some studies. Although 18 (66.7%) patients were classified as grade 3 in our cohort, the small size needs expansion to evaluate OX-CT efficacy in G3 NETs in the future. Fourthly, data concerning adverse events were not reported in detail in many included studies, so did our cohort. The toxicities of oxaliplatin-based chemotherapy in NETs were consistent with the previously published safety profiles (57). A drug holiday in patients with stable or responding disease after envisaged or actual cycles might be suitable. Two patients in our cohort had a treatment-free interval of 9.3 and 19.7 m, respectively.

## Conclusion

**Figure 6:**
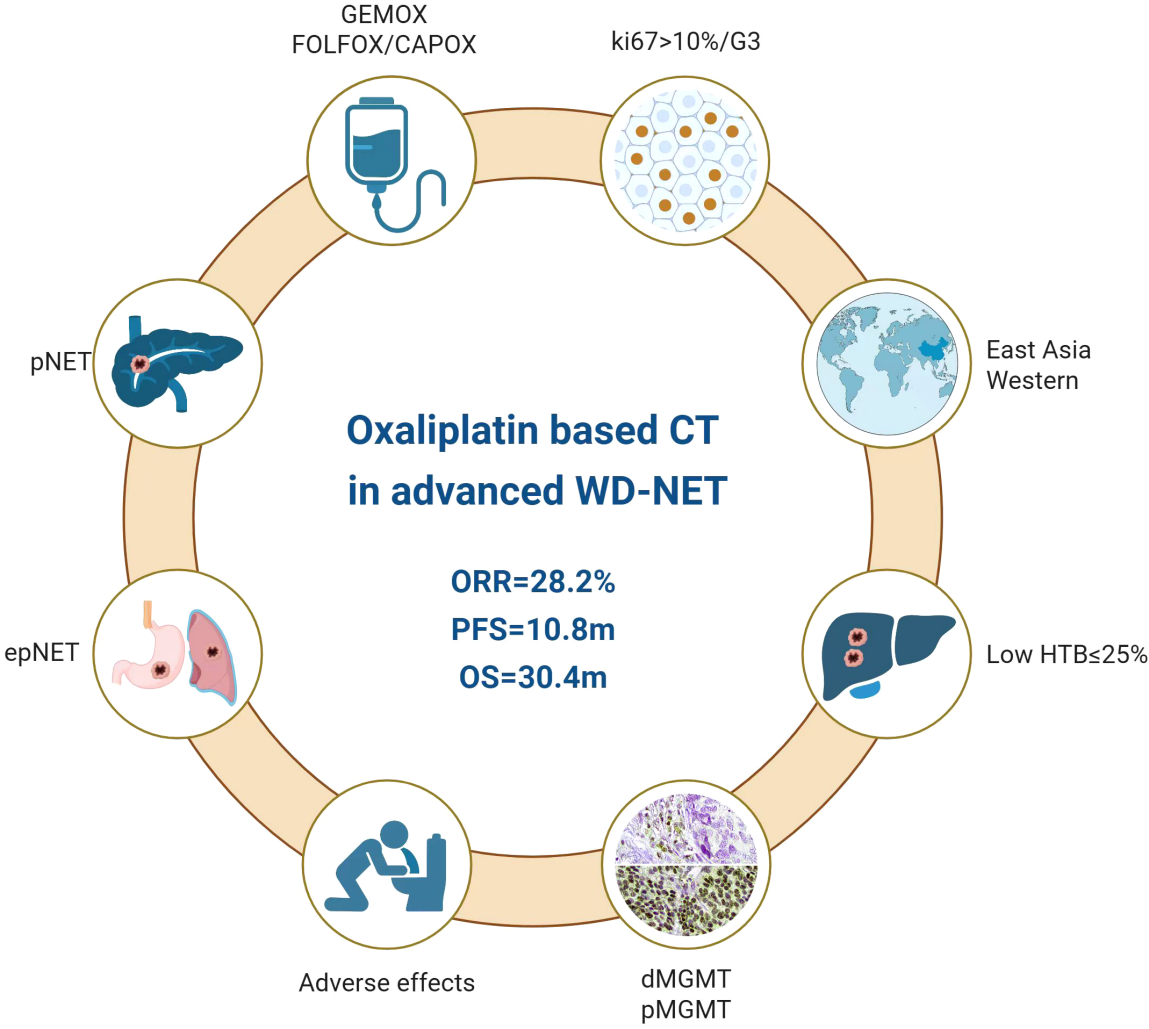
Roles of oxaliplatin-based chemotherapy in advanced well-differentiated neuroendocrine tumors.

Oxaliplatin-based chemotherapy was shown to be an effective and well-tolerated therapeutic option in advanced well differentiated neuroendocrine tumors, as evidenced by both the systemic review and our small Chinese cohort (**Figure 6**). Notably, the efficacy was independent of MGMT status and specific chemotherapy regimens (GEMOX or fluoropyrimidine-based chemotherapy). A favorable response was seen in NETs of pancreatic origin, those with high Ki-67 more than 10% and low hepatic tumor burden (≤25%). In the future, the personalized approach to oxaliplatin-based chemotherapy will be largely based on both clinical and molecular-driven thinking within a NEN-dedicated multidisciplinary team.

## Data Availability

The original contributions presented in the study are included in the article/**Supplementary Material**. Further inquiries can be directed to the corresponding author.
