# Supplementary material for: Predictive factors for efficacy of oxaliplatin-based chemotherapy in advanced well-differentiated neuroendocrine tumors: an observational cohort study and meta-analysis
Source: Front Endocrinol (Lausanne). 2025 May 14;16:1595151. doi: 10.3389/fendo.2025.1595151 (PMC12116336; doi:10.3389/fendo.2025.1595151)
Supplement: Supplementary file 2 [file DataSheet2.docx]

Figure S1 Flowchart of study selection process according to PRISMA statement

Figure S2 Forest plots representing the meta-analysis of ORR and DCR in different subgroup: A, ORR of all patients; B, ORR of pNET and epNET; C, ORR of G3 and G1-2 NETs; D, DCR of all patients.

Figure S3. Forest plots representing the meta-analysis of ORR and PFS in pNET and epNET、G3 NET and G1-2 NET subgroup after the studies with scores of 6 or lower were excluded using JBI PACES: A, ORR of pNET and epNET; B, PFS of pNET and epNET; C, ORR of G3 NET and G1-2 NET; D, PFS of G3 NET and G1-2 NET.

Figure S4. Forest plots representing the meta-analysis of median PFS (months) and OS (months) in different subgroup: A, PFS of all patients; B, PFS of pNET and epNET; C, PFS of G3 and G1-2 NETs; D, OS of all patients.

Figure S5. Forest plots representing the meta-analysis of ORR and PFS in GEMOX and FOLFOX/CAPOX chemotherapy in the meta-analysis: A, ORR; B, PFS.

Figure S6. Forest plots representing the meta-analysis of ORR and PFS in GEMOX and FOLFOX/CAPOX chemotherapy in thoracic NETs in the meta-analysis: A, ORR; B, PFS.
