# Supplementary figures and images for: Predictive factors for efficacy of oxaliplatin-based chemotherapy in advanced well-differentiated neuroendocrine tumors: an observational cohort study and meta-analysis
Source: Front Endocrinol (Lausanne). 2025 May 14;16:1595151. doi: 10.3389/fendo.2025.1595151 (PMC12116336; doi:10.3389/fendo.2025.1595151)

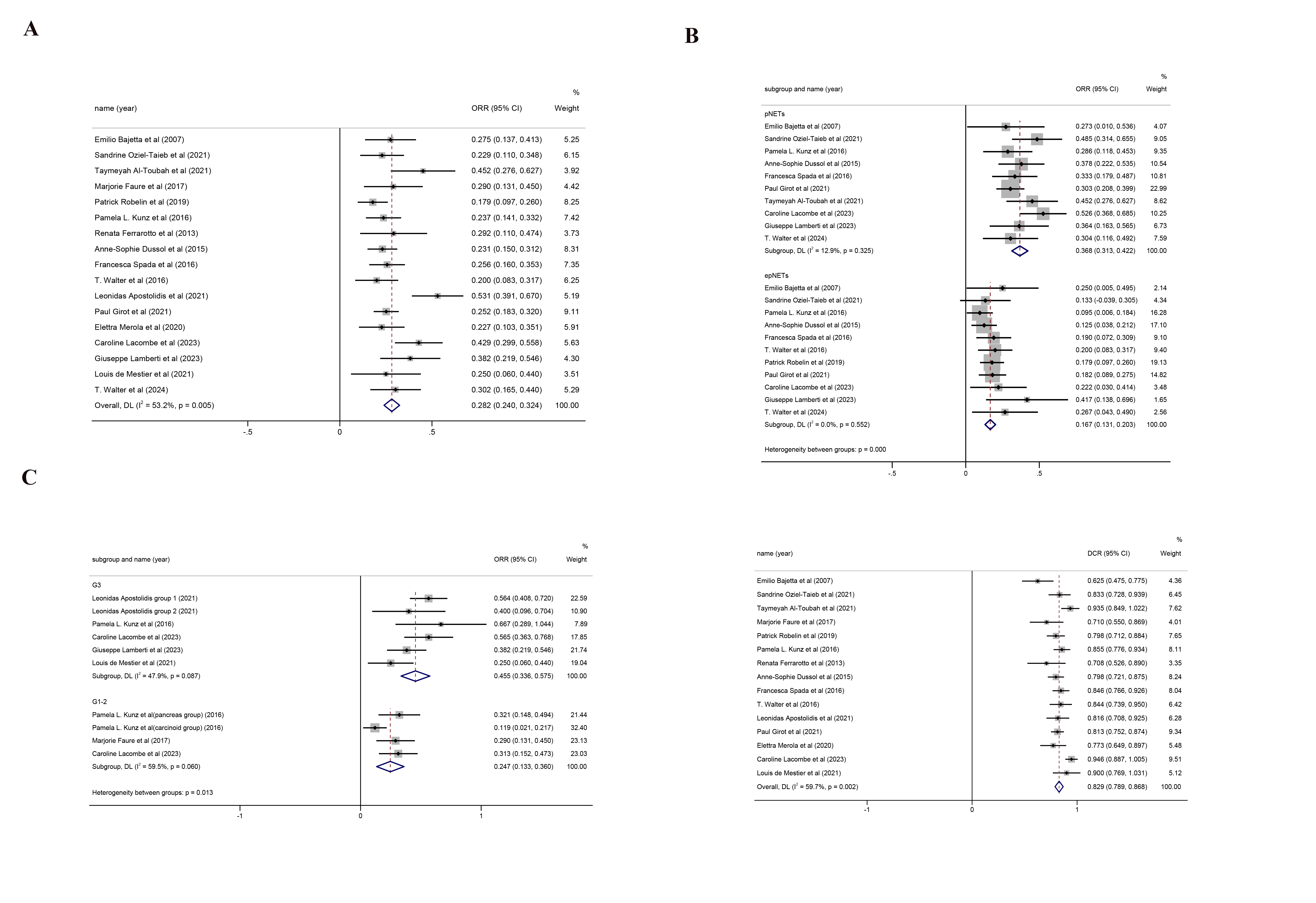

Supplement: Supplementary file 4 [file Image2.jpeg]

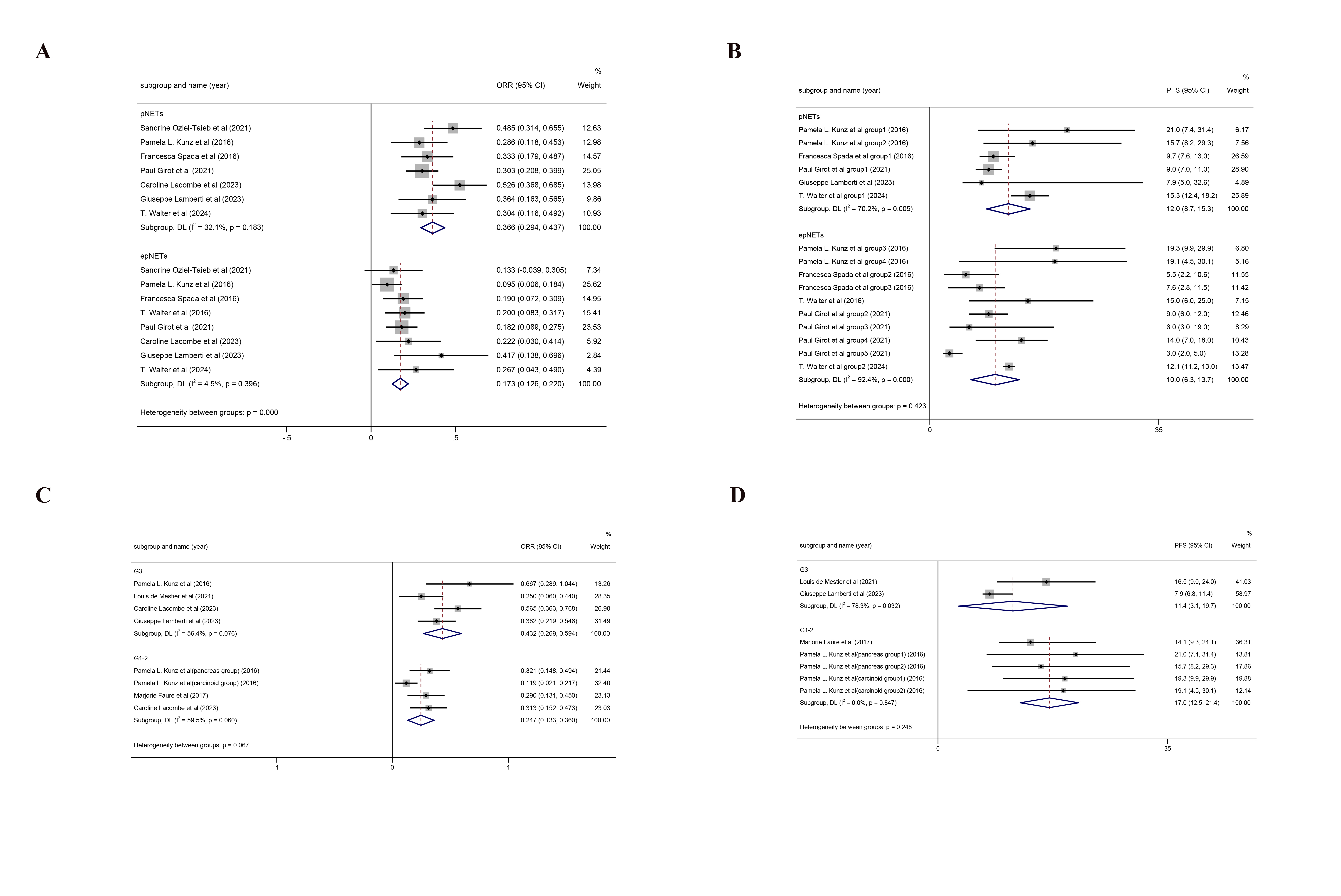

Supplement: Supplementary file 5 [file Image3.jpeg]

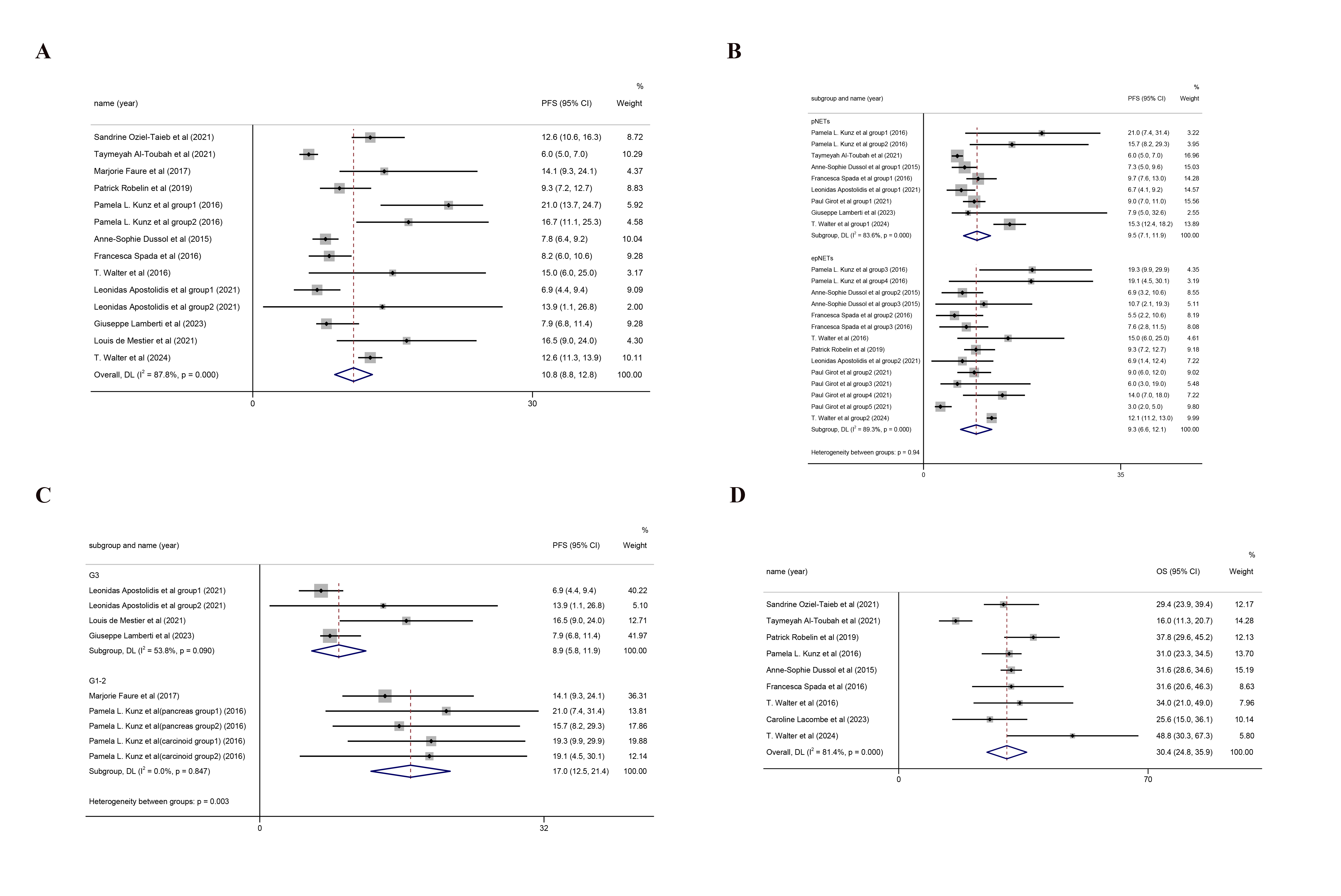

Supplement: Supplementary file 6 [file Image4.jpeg]

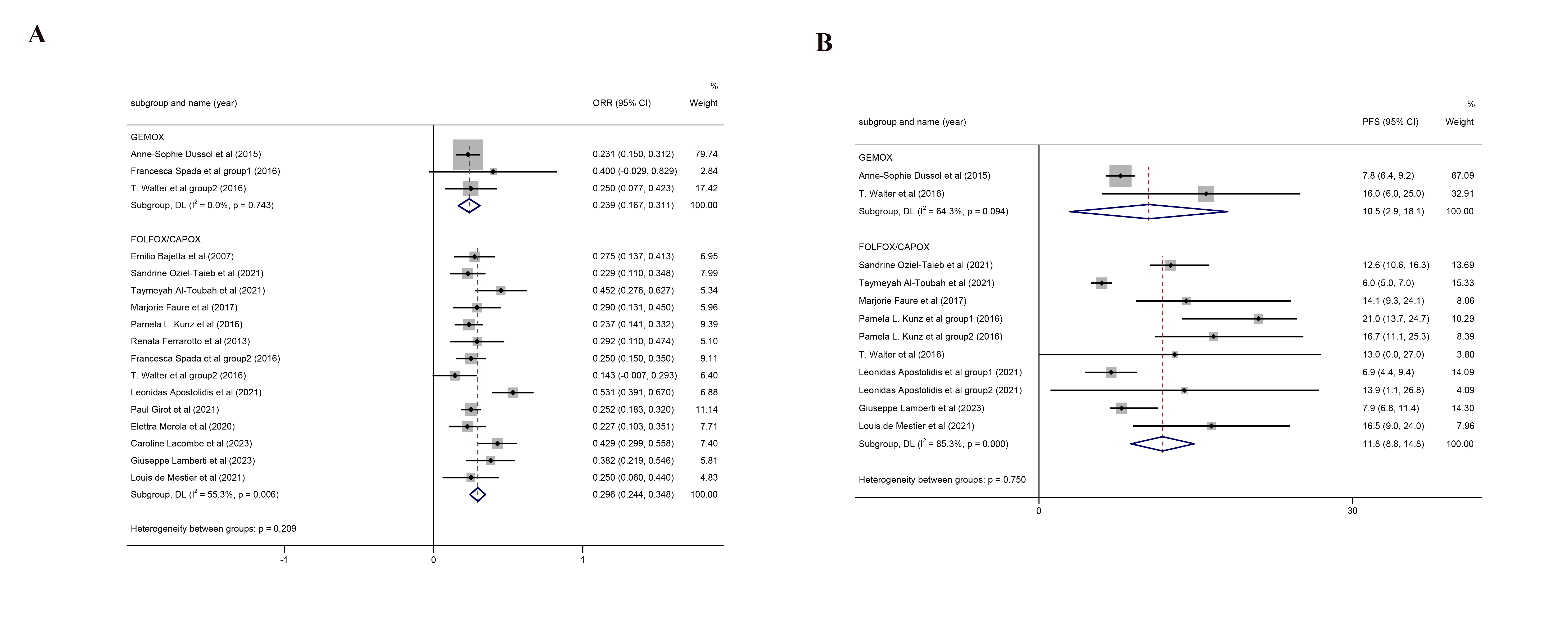

Supplement: Supplementary file 7 [file Image5.jpeg]

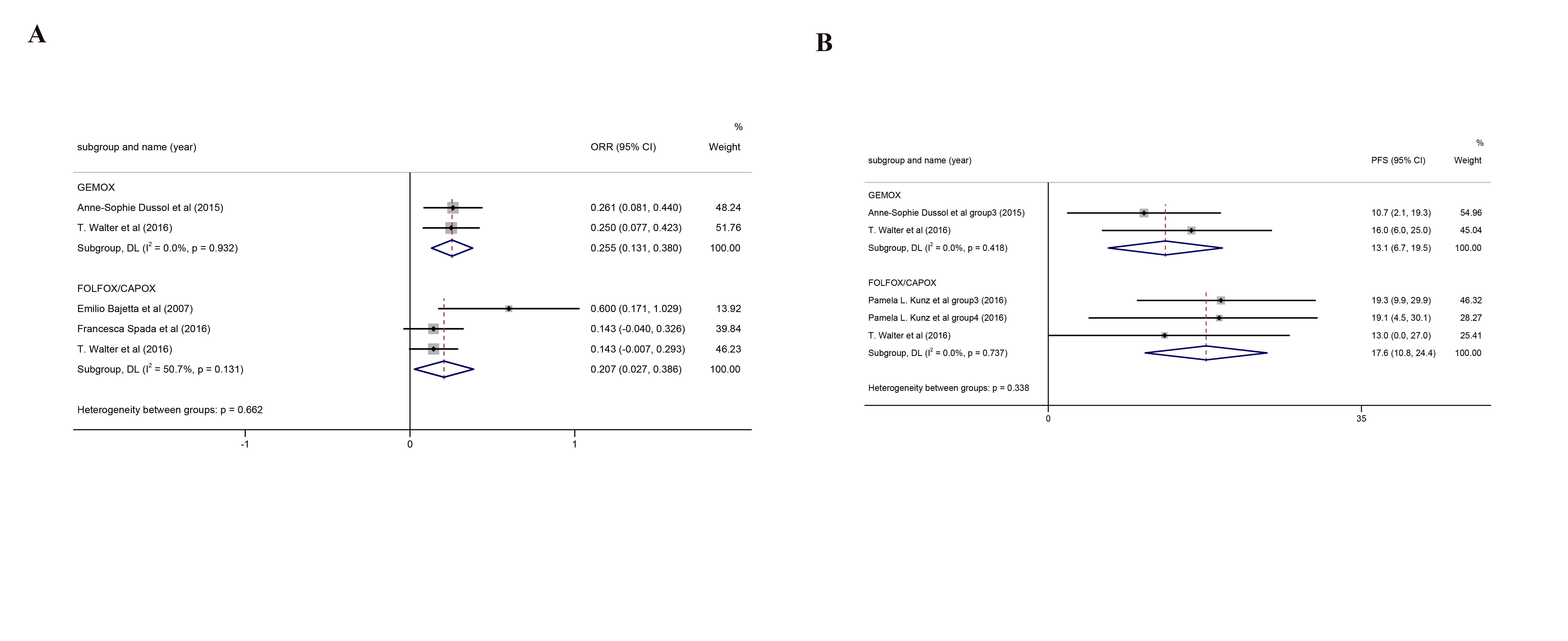

Supplement: Supplementary file 8 [file Image6.jpeg]
