# Supplementary material for: Predictive factors for efficacy of oxaliplatin-based chemotherapy in advanced well-differentiated neuroendocrine tumors: an observational cohort study and meta-analysis
Source: Front Endocrinol (Lausanne). 2025 May 14;16:1595151. doi: 10.3389/fendo.2025.1595151 (PMC12116336; doi:10.3389/fendo.2025.1595151)
Supplement: Supplementary file 9 [file Table1.docx]

**supplementary Table 1. Quality evaluation of included studies using JBI PACES**

| Study | Q1 | Q2 | Q3 | Q4 | Q5 | Q6 | Q7 | Q8 | Q9 | Score | Ref |
| --- | --- | --- | --- | --- | --- | --- | --- | --- | --- | --- | --- |
| Emilio Bajetta et al, 2007 | Y | Y | U | U | Y | Y | Y | N | Y | 6 | ^34^ |
| Sandrine Oziel-Taieb et al, 2021 | Y | Y | U | Y | Y | Y | U | Y | Y | 7 | ^21^ |
| Taymeyah Al-Toubah et al, 2021 | Y | Y | U | U | Y | Y | Y | Y | U | 6 | ^22^ |
| Marjorie Faure et al, 2017 | Y | Y | U | Y | Y | Y | Y | Y | U | 7 | ^23^ |
| Patrick Robelin et al, 2019 | Y | Y | U | U | Y | Y | U | Y | Y | 6 | ^24^ |
| Pamela L. Kunz et al, 2016 | Y | Y | Y | U | Y | Y | Y | Y | N | 7 | ^33^ |
| Renata Ferrarotto et al, 2013 | Y | U | U | N | Y | Y | Y | Y | Y | 6 | ^30^ |
| Anne-Sophie Dussol et al, 2015 | Y | U | U | N | Y | Y | Y | Y | Y | 6 | ^11^ |
| Francesca Spada et al, 2016 | Y | Y | U | Y | Y | Y | U | Y | Y | 7 | ^25^ |
| Thomas Walter et al, 2016 | Y | Y | U | Y | Y | Y | U | Y | Y | 7 | ^26^ |
| Leonidas Apostolidis et al, 2021 | Y | Y | U | U | Y | Y | N | Y | Y | 6 | ^27^ |
| Paul Girot et al, 2021 | Y | Y | U | Y | Y | Y | N | Y | Y | 7 | ^31^ |
| Elettra Merola et al, 2020 | Y | Y | U | Y | Y | Y | N | Y | Y | 7 | ^32^ |
| Caroline Lacombe et al, 2022 | Y | Y | U | Y | Y | Y | Y | Y | Y | 8 | ^14^ |
| Giuseppe Lamberti et al, 2023 | Y | Y | U | N | Y | Y | Y | Y | Y | 7 | ^28^ |
| Louis de Mestier et al, 2021 | Y | Y | U | Y | Y | Y | Y | Y | Y | 8 | ^29^ |
| Thomas Walter et al, 2024 | Y | Y | U | Y | Y | Y | Y | Y | Y | 8 | ^13^ |

N, no; U, unclear; Y, yes.

Q1: Was the sample frame appropriate to address the target population?

Q2: Were study participants sampled in an appropriate way?

Q3: Was the sample size adequate?

Q4: Were the study subjects and the setting described in detail?

Q5: Was the data analysis conducted with sufficient coverage of the identified sample?

Q6: Were valid methods used for the identification of the condition?

Q7: Was the condition measured in a standard, reliable way for all participants?

Q8: Was there appropriate statistical analysis?

Q9: Was the response rate adequate, and if not, was the low response rate managed appropriately?

Studies that scored five or more ‘‘Yes’’ ratings out of nine were included in the meta-analysis.

**supplementary Table 2. Summary of the selected studies**

| Study | Study design | Treatment regimens | Cycles(n) | Patients  (n) | Treatment lines | Tumor sites | | WHO grade | ORR | DCR | mPFS  (months) | mOS  (months) | Ref |
| --- | --- | --- | --- | --- | --- | --- | --- | --- | --- | --- | --- | --- | --- |
| Emilio et al, 2007 | Phase II  prospective | CAPOX | 1-6 | 40 | first line (32.5%)  second line (67.5%) | | pNETs (37.5%) epNETs (62.5%) | NA | 27.5%  (11/40) | 62.5%  (25/40) | NA | 32 | ^34^ |
| Sandrine et al, 2021 | retrospective | FOLFOX | 6(2-12) | 48 | first line (58.3%)  ≥second line (41.7%) | | pNETs (68.8%) epNETs (31.2%) | G1/G2/G3 | 22.9%  (11/48) | 83.3%  (40/48) | 12.6 | 29.4 | ^21^ |
| Taymeyah et al, 2021 | retrospective | FOLFOX± bevacizumab | NA | 31 | ≥second line | | pNETs | G1/G2/G3 | 45.2%  (14/31) | 93.5%  (29/31) | 6 | 16 | ^22^ |
| Marjorie et al, 2017 | retrospective | FOLFOX | 6(1-22) | 31 | first line (58.1%)  ≥second line (41.9%) | | pNETs (45.2%) epNETs (54.8%) | G1/G2 | 29%  (9/31) | 71%  (22/31) | 14.1 | NA | ^23^ |
| Patrick et al, 2019 | retrospective | Oxaliplatin-Based | 8(1-24) | 84 | first line (26.2%)  ≥second line (73.8%) | | epNETs | G1/G2 | 17.9%  (15/84) | 79.8%  (67/84) | 9.3 | 37.8 | ^24^ |
| Pamela et al, 2016 | Phase II  prospective | FOLFOX/CAPOX+ bevacizumab | 17  (3-51) | 76 | NA | | pNETs (36.8%) epNETs (63.2%) | G1/G2/G3 | 23.7%  (18/76) | 85.5%  (65/76) | NA | NA | ^33^ |
| Renata et al, 2013 | retrospective | CAPOX | 6(2-13) | 24 | first line (50.0%)  ≥second line (50.0%) | | pNETs (62.5%) epNETs (37.5%) | G1/G2/G3 | 29%  (7/24) | 71%  (17/24) | 9.8 | NA | ^30^ |
| Anne et al, 2015 | retrospective | GEMOX | 6(1-10) | 104 | first line (20.2%)  ≥second line (79.8%) | | pNETs (35.6%) epNETs (64.4%) | G1/G2/G3 | 23% (24/104) | 86% (83/104) | 7.8 | 31.6 | ^11^ |
| Francesca et al, 2016 | retrospective | GEMOX/CAPOX/FOLFOX | 4.5  (1-9) | 78 | first line (12.8%)  ≥second line (87.2%) | | pNETs (46.2%) epNETs (53.8%) | G1/G2/G3 | 26%  (20/78) | 90%  (66/78) | 8.2 | 31.6 | ^25^ |
| T. Walter et al, 2016 | retrospective | GEMOX/FOLFOX | 8 | 45 | first line (20.0%)  ≥second line (80.0%) | | epNETs | N/A | 20%  (9/45) | 84%  (38/45) | 15.0 | 34.0 | ^26^ |
| Leonidas et al, 2021 | retrospective | FOLFOX | NA | 49 | first line (79.6%)  second line (20.4%) | | pNETs (79.5%) epNETs (20.5%) | G3 | 53.1%  (26/49) | 81.6%  (40/49) | NA | NA | ^27^ |
| Paul et al, 2021 | retrospective | FOLFOX | 8(3-28) | 155 | first line (32.3%)  ≥second line (67.7%) | | pNETs (57.4%) epNETs (42.6%) | G1/G2/G3 | 25.2% (39/155) | 81.3%(126/155) | 9 | 28 | ^31^ |
| Elettra et al, 2020 | retrospective | FOLFOX | 7(2-21) | 44 | first line (22.7%)  ≥second line (77.3%) | | N/A | G1/G2/G3 | 22.7%  (10/44) | 77.3%  (30/44) | NA | NA | ^32^ |
| Caroline et al, 2023 | retrospective | FOLFOX+ bevacizumab | 6(5-10) | 56 | first line (17.5%)  ≥second line (82.5%) | | pNETs (66.7%) epNETs (33.3%) | G1/G2/G3 | 42.9%  (24/56) | 94.6%  (53/56) | NA | 25.6 | ^14^ |
| Giuseppe et al, 2023 | retrospective | CAPOX/FOLFOX | NA | 34 | first line | | pNETs (64.7%) epNETs (35.3%) | G3 | 38.2%  (13/34) | NA | 7.9 | 30 | ^28^ |
| Louis et al, 2023 | retrospective | Oxaliplatin-Based | NA | 20 | first line (40%)  ≥second line (60%) | | NA | G3 | 25%  (5/20) | 90%  (18/20) | 16.5 | NA | ^29^ |
| T. Walter et al, 2024 | Phase II  prospective | GEMOX/CAPOX/FOLFOX | 8(6-10) | 43 | NA | | pNETs (51.1%) epNETs (48.9%) | G1/G2/G3 | 30.2%  (13/43) | NA | 12.6 | 48.8 | ^13^ |

FOLFOX: oxaliplatin plus 5-Fluorouracil, CAPOX: oxaliplatin plus capecitabine, GEMOX: gemcitabine plus oxaliplatin, pNETs: pancreatic NETs, epNETs: extra-pancreatic NETs.

**supplementary Table 3. Summary of Grade 3 and higher adverse effects in the 12 studies**

| **Ref** | **Patients**  **(N)** | **Any grade AE**  **N (%)** | **≥G3 AE**  **N (%)** | **Gastrointestinal ≥G3 N (%)** | **Hematological  ≥G3 N (%)** | **Neurotoxicity ≥G3 N (%)** | **HFS**  **≥G3 N (%)** | **Fatigue**  **≥G3 N (%)** | **Hypertension^&^**  **≥G3 N (%)** | **Nephrotoxicity^&^**  **≥G3 N (%)** | **Others   ≥G3 N (%)** |
| --- | --- | --- | --- | --- | --- | --- | --- | --- | --- | --- | --- |
| ^34^ | 40 | NA | 4 (10) | 1 (2.5) | NA | NA | NA | 3 (7.5) | NA | NA | NA |
| ^21^ | 48 | 1 | 1 (2.1) | NA | NA | NA | NA | NA | NA | NA | 1 (2.1) |
| ^22^ | 31 | 20 | 8 (25.8) | 1(3.2) | 1 (3.2) | 2 (6.5) | NA | 1 (3.2) | NA | NA | 3 (9.7) |
| ^23^ | 31 | 17 | NA | NA | NA | NA | NA | NA | NA | NA | NA |
| ^24^ | 84 | 22 | NA | NA | NA | NA | NA | NA | NA | NA | NA |
| ^33^ | 76 | NA | 38 (50.0) | 21 (27.6) | 18 (23.7) | 9 (11.8) | 4 (5.3) | 7 (9.2) | 9 (11.8) | 2 (2.6) | 19 (25.0) |
| ^30^ | 24 | 21 | 6 (25.0) | 2 (8.3) | NA | 2 (8.3) | 1 (4.2） | NA | NA | NA | 1 (4.2） |
| ^25^ | 78 | NA | NA | 5 (6.0) | 2 (3.0) | 8 (10.0) | NA | NA | NA | NA | 2 (3.0) |
| ^26^ | 45 | 8 | 8 (18.0) | NA | NA | 1 (2.2) | NA | 3 (6.7) | NA | NA | 4 (8.9) |
| ^31^ | 155 | NA | NA | NA | 2 (1.2) | 28 (18.0) | NA | NA | NA | NA | 4 (2.6) |
| ^32^ | 72 | 64 | NA | NA | 7 (9.7) | 3 (4.2) | NA | NA | NA | NA | 12 (16.7) |
| ^14^ | 57 | 54 | NA | 2 (3.5) | 8 (14.0) | 24 (44.4) | 3 (5.6) | 7 (12.3) | 6 (11.1) | NA | 6 (11.1) |
| ^13^ | 43 | 43 | 18 (41.9) | 4 (9.3) | 5 (11.6) | NA | NA | 1 (1.3) | NA | NA | 5 (11.6) |
| Total | 784 | 250 (54.5) | 83 (24.2) | 36 (7.6) | 43 (8.1) | 76 (11.8) | 8 (6.1) | 22 (6.0) | 15 (11.2) | 2 (2.6) | 57 (8.1) |
| 95% CI | | (21.0-88.1) | (9.4-38.9) | (2.8-12.5) | (3.3-12.8) | (5.5-18.2) | (3.1-9.2) | (2.9-9.0) | (5.9-16.6) | (NA) | (4.3-11.8) |

NA, non-available; HFS: hand food syndrome; Cl = confidence interval.

^&^, hypertension and nephrotoxicity were only reported in those patients treated with bevacizumab.
